# A new rapid method for direct antimicrobial susceptibility testing of bacteria from positive blood cultures

**DOI:** 10.1186/s12866-016-0805-5

**Published:** 2016-08-12

**Authors:** Simona Barnini, Veronica Brucculeri, Paola Morici, Emilia Ghelardi, Walter Florio, Antonella Lupetti

**Affiliations:** 1 Azienda Ospedaliero-Universitaria Pisana, Pisa, Italy; 2 Dipartimento di Ricerca Traslazionale e delle Nuove Tecnologie in Medicina e Chirurgia, Università di Pisa, Pisa, Italy

**Keywords:** Bloodstream infection, Blood culture, Direct AST, Bactec FX, Alfred 60AST, MALDI-TOF

## Abstract

**Background:**

Rapid identification and antimicrobial susceptibility testing (AST) of the causative agent(s) of bloodstream infections can lead to prompt appropriate antimicrobial therapy. To shorten species identification, in this study bacteria were recovered from monomicrobial blood cultures by serum separator tubes and spotted onto the target plate for direct MALDI-TOF MS identification. Proper antibiotics were selected for direct AST based on species identification. In order to obtain rapid AST results, bacteria were recovered from positive blood cultures by two different protocols: by serum separator tubes (further referred to as PR1), or after a short-term subculture in liquid medium (further referred to as PR2). The results were compared with those obtained by the method currently used in our laboratory consisting in identification by MALDI-TOF and AST by Vitek 2 or Sensititre on isolated colonies.

**Results:**

The direct MALDI-TOF method concordantly identified with the current method 97.5 % of the Gram-negative bacteria and 96.1 % of the Gram-positive cocci contained in monomicrobial blood cultures. The direct AST by PR1 and PR2 for all isolate/antimicrobial agent combinations was concordant/correct with the current method for 87.8 and 90.5 % of Gram-negative bacteria and for 93.1 and 93.8 % of Gram-positive cocci, respectively. In particular, 100 % categorical agreement was found with levofloxacin for Enterobacteriaceae by both PR1 and PR2, and 99.0 and 100 % categorical agreement was observed with linezolid for Gram-positive cocci by PR1 and PR2, respectively. There was no significant difference in accuracy between PR1 and PR2 for Gram-negative bacteria and Gram-positive cocci.

**Conclusions:**

This newly described method seems promising for providing accurate AST results. Most importantly, these results would be available in a few hours from blood culture positivity, which would help clinicians to promptly confirm or streamline an effective antibiotic therapy in patients with bloodstream infections.

**Electronic supplementary material:**

The online version of this article (doi:10.1186/s12866-016-0805-5) contains supplementary material, which is available to authorized users.

## Background

Rapid identification (ID) and antimicrobial susceptibility testing (AST) of the causative agent(s) of bloodstream infections (BSI) are essential for the timely selection of an appropriate antimicrobial therapy, which may result in a better outcome for patients [1–4], and contribute to contrast the emergence of antimicrobial resistance [5].

The current method requires that a positive blood culture (BC) is subcultured onto solid media before ID and AST are performed by automated systems. To shorten the turnaround time for diagnosis, several methods have been proposed and evaluated, some of which with promising results [6–21].

In the present study, we aimed at establishing a method able to provide rapid AST results, further referred to as direct AST. To this aim, we used a CE approved system, named Alfred 60AST (Alifax), initially conceived for urine screening, and for AST of bacterial isolates from urine [22]. In order to adapt the system to rapid AST from positive blood cultures, two different protocols, protocol 1 (PR1) and protocol 2 (PR2), were evaluated, the first using bacteria harvested from positive BC by serum separator tubes, the second using a short-term subculture of a positive BC in liquid medium. MALDI-TOF identification of bacteria contained in monomicrobial BC was performed by a recently described method, further referred to as direct MALDI-TOF method [11]. The ID results obtained by the direct MALDI-TOF method and the AST results obtained by PR1 and PR2 were compared with those by the method currently used in our laboratory, further referred to as current method, i.e. ID and AST on isolated colonies by MALDI-TOF and Vitek 2 or Sensititre, respectively.

## Methods

### Blood samples

Blood specimens from patients admitted to the Pisa University Hospital (Italy) in the period July-December 2014 were inoculated into BC bottles [Plus Aerobic/F and Plus Anaerobic/F, or Peds Plus F (Becton Dickinson & Co, BD, Milan, Italy)], collected at the Unità Operativa di Microbiologia, and transferred to the Bactec FX instrument (Becton Dickinson, Franklin Lakes, NJ, USA) for monitoring bacterial growth. From each patient, only the first positive BC apparently monomicrobial at the Gram staining was included in this study.

### Identification and AST of bacteria by the current method

BC positive at the Bactec FX instrument were subjected to Gram staining and subcultured onto appropriate solid media. Isolated colonies were identified by MALDI-TOF (Bruker Daltonics, Bremen, Germany) and AST was performed by Vitek 2 (advanced Expert System software, version R04.02C; bioMérieux, Marcy l’Étoile, France) or, for *Acinetobacter baumannii* and *Stenotrophomonas maltophilia* isolates, by the Sensititre Aris system (Trek Diagnostic Systems, Thermo Fisher Scientific, OH, USA). The ID and AST results by the current method were used as comparator for evaluation of results obtained by PR1 and PR2.

Identification and AST cards were tested periodically for quality control assessment.

### Identification of bacteria by the direct MALDI-TOF method

Identification of bacteria by the direct MALDI-TOF method was performed as previously described [11]. MALDI-TOF analysis was performed using a Microflex LT system mass spectrometer following the manufacturer’s instructions. Captured spectra were analyzed using a MALDI-TOF Biotyper automated control and the Bruker Biotyper 3.1 software and library (4624 isolates) (Bruker Daltonics). For each plate, the instrument was calibrated to validate the run.

Identification results by the direct MALDI-TOF method were compared with those by MALDI-TOF on isolated colonies, as reference. Discrepancies in identification were resolved by Vitek 2.

### Direct AST of bacteria

Direct AST of bacteria was performed by Alfred 60AST (Alifax SpA, Polverara, PD, Italy) and carried out using two different protocols, PR1 and PR2. By PR1, bacteria were recovered from positive BC by serum separator tubes (BD) as previously described [11]. Briefly, an eight-ml sample of a positive blood culture was transferred to serum separator tubes. Next, bacteria were sedimented on the surface of the silicon layer of the vacutainer tube by centrifugation at 2,000 × g for 10 min, and suspended at 0.9 McFarland in vials containing 2 ml HB&L broth (Alifax). The bacterial suspension was transferred into an AST-Empty vial and loaded in the refrigerated area dedicated to external samples, called “External Samples Zone” of the Alfred 60AST system. By PR2, 20 μl of positive BC were inoculated into 2 ml HB&L broth and loaded in the thermostat of the Alfred 60AST system for monitoring the bacterial growth up to 0.5 McFarland. Next, the instrument automatically transferred the sample into an AST-Empty vial, placed in the refrigerated area “Alfred Sample Zone”. The time needed to reach a bacterial density corresponding to 0.5 McFarland was 1–4 h, depending on the bacterial species (6 h for *S. maltophilia*).

Lyophilized antibiotics (Alifax) were dissolved in 2 ml regenerating solution, and stored at 4 °C for up to 6 days (3 days for meropenem). The regenerated antibiotics were loaded into the “Alfred Antibiotic Zone”. The antibiotics tested were: levofloxacin and gentamicin for all Gram-negative bacteria; cefotaxime, ceftazidime and meropenem for Enterobacteriaceae, and amikacin and colistin for nonfermenters; linezolid and teicoplanin for all Gram-positive cocci; cefoxitin for staphylococci, and ampicillin for streptococci.

For each strain a panel of antibiotics was established on the basis of antibiotics available for this system at the time we started this study, and on the basis of the time needed to perform the test. We preferably selected antibiotics requiring three hours incubation instead of five. Each antibiotic was CE approved. The vials, each containing 2 ml HB&L broth, were put in the thermostat, and automatically loaded with the bacterial suspension (100 μl) and the selected antibiotic (200 μl) for both PR1 and PR2. One vial was loaded only with the bacterial suspension and used as a positive control for bacterial growth.

### Interpretation of AST results and data analyses

The antimicrobial activity of the tested antibiotics (by PR1 and PR2) was calculated by the system as percentage of bacterial growth in comparison to the positive control. The percentage of bacterial growth was automatically translated into clinical categories: a growth between 100 and 65 % was interpreted as resistant, between 65 and 50 % as intermediate, and < 50 % as susceptible (EUCAST-defined breakpoints [23]). The clinical categories obtained by the different methods were compared with the results by the current method and expressed as: agreement, very major errors (false susceptible), major errors (false resistant), and minor errors (susceptible/resistant versus intermediate susceptibility). Discrepancies between the results by direct AST (PR1 or PR2) and the current method were resolved by Etest (AB BioMerieux), which was performed according to the manufacturer’s instructions.

The percentages of very major errors and major errors were calculated according to the current ISO 20776–2 guideline. Major error rate was calculated based on the number of susceptible strains tested as denominator; very major error rate was calculated based on the number of resistant strains tested as denominator.

Statistical analysis was performed using the chi-square test for independent pairs. The level of significance was set at *P* < 0.05.

## Results

### Identification of bacteria from positive BC by direct MALDI-TOF

A total of 194 positive BC were apparently monomicrobial at the Gram staining, eleven (5.7 %) of which resulted to be polymicrobial after subculture and were excluded from this study. All monomicrobial BC contained either Gram-negative bacilli or Gram-positive cocci. Eighty monomicrobial BC containing Gram-negative bacteria and 103 containing Gram-positive cocci were processed by the direct MALDI-TOF method, with a mean identification time of 25 min after BC positivity. In parallel, BC were subcultured onto agar media for microbial identification by the current method.

Identification of Gram-negative bacteria by the direct MALDI-TOF method gave an interpretable result for 78 (97.5 %) of 80 BC (Table 1), 73 (91.3 %) of which with scores > 2.0. The unidentified strains were correctly identified by MALDI-TOF the day after on isolated colonies. Duplicates were 100 % concordant and gave similar scores. No strain was misidentified by the direct MALDI-TOF method. Identification of bacteria by the direct and current methods by MALDI-TOF was always concordant.

**Table 1:** Gram-negative bacteria from monomicrobial blood cultures identified by the direct MALDI-TOF method

Species	Correctly identified with score value of:				Unidentified	Total
	3.000–2.300	2.299–2.000	1.999–1.700	<1.700		
*Escherichia coli*	17	7				24
*Klebsiella pneumoniae*	12	11	1		1	25
*Enterobacter aerogenes*	1	1				2
*Enterobacter cloacae*		4				4
*Morganella morganii*	1	1				2
*Serratia marcescens*		1	1			2
*Salmonella spp.*		1				1
*Proteus mirabilis*	1					1
*Raoultella ornithinolytica*	1					1
*Pseudomonas aeruginosa*	5	3	2	1	1	12
*Acinetobacter baumannii*		3				3
*Stenotrophomonas maltophilia*		2				2
*Acinetobacter ursingii*		1				1
Total	38	35	4	1	2	80

Identification of Gram-positive cocci by the direct and current methods by MALDI-TOF gave concordant results for 99 (96.1 %) of the 103 BC, although only 45 (43.7 %) with scores ≥ 2.0 (Table 2). Duplicates were 100 % concordant and gave similar scores.

**Table 2:** Gram-positive cocci from monomicrobial blood cultures identified by the direct MALDI-TOF method

Species	Correctly identified with score value of:				Unidentified	Misidentified	Total
	3.000–2.300	2.299–2.000	1.999–1.700	<1.700			
*Staphylococcus epidermidis*		8	21	13			42
*Staphylococcus aureus*		12	3	1	1		17
*Staphylococcus hominis*		5	4	2	1	1	13
*Staphylococcus capitis*	1	9	1				11
*Staphylococcus haemolyticus*		4		1			5
*Staphylococcus warneri*			1	1			2
*Staphylococcus pettenkoferi*			1				1
*Staphylococcus sciuri*		1					1
*Staphylococcus lugdunensis*			1				1
*Enterococcus faecium*	1	2	1				4
*Enterococcus faecalis*	1	1	1				3
*Enterococcus casseliflavus*			1				1
*Streptococcus sanguinis*				1			1
*Streptococcus pneumoniae*						1	1
Total	3	42	35	19	2	2	103

One *Staphylococcus hominis* was erroneously identified by the direct method as *Staphylococcus haemolyticus*, and one *Streptococcus pneumoniae* as *Streptococcus oralis* group mitis. Furthermore, one *S. hominis* and one *Staphylococcus aureus* strains were unidentified by the direct method and correctly identified by MALDI-TOF on isolated colonies.

### Direct AST of Gram-negative bacilli

The AST results by PR1 and/or PR2 were available for all the 80 Gram-negative isolates tested, 62 of which were Enterobacteriaceae (Table 3) and 18 nonfermenters (Table 4). The number of resistant, intermediate, and sensitive strains for each antibiotic according to the current method is reported as additional material in (Additional file 1: Table S1). The AST by PR1 failed for 6 isolates (1 *Enterobacter cloacae*, 2 *P. aeruginosa*, and 3 *A. baumannii*), and by PR2 for other 6 (1 *K. pneumoniae*, 1 *Serratia marcescens*, 1 *Acinetobacter ursingii*, 1 *P. aeruginosa*, and 2 *S. maltophilia*). Overall, the antimicrobial susceptibility testing of Gram-negative bacilli (Tables 3 and 4) was assessed for a total of 353 (PR1) and 348 (PR2) isolate/antimicrobial agent combinations. Discrepancies between the direct and current methods were resolved by Etest, which revealed that AST was correct by PR1 or PR2, but not by the current method, for 15 (4.2) and 9 (2.6 %) isolate/antimicrobial agent combinations, respectively, all of which, but one, regarding Enterobacteriaceae. Complete AST agreement (i.e., AST agreement for all tested antibiotics) was found for 35 (43.7) and 38 (47.5 %) out of the 80 isolates by PR1 and PR2, respectively. The AST results of Gram-negative bacilli and the total percent error as well as the percent error for each antimicrobial agent are shown in Tables 3 and 4. Together, concordant/correct AST for Gram-negative bacteria was found for 87.8 and 90.5 % of all isolate/antimicrobial combinations by PR1 and PR2, respectively. Notably, AST agreement was higher than 90 % for cefotaxime, levofloxacin, and amikacin by PR1, and for levofloxacin, ceftazidime, amikacin, and colistin by PR2. In the overall, no significant difference in accuracy was found between PR1 and PR2 for Gram-negative bacteria (0.2 < *P* < 0.5).

**Table 3:** Antimicrobial susceptibility testing of Enterobacteriaceae from positive blood cultures by PR1 (A)^a^ and PR2 (B)^a^

Antimicrobial agent	No. of very major errors	No. of major errors	No. of minor errors	AST agreement	Total
A					
Cefotaxime (CTX)	0/40	1/19 (5.3 %)	2/59 (3.4 %)	56 (94.9 %)	59
Ceftazidime (CAZ)	0/32	10/25 (40 %)	4/60 (6.7 %)	46 (76.7 %)	60
Gentamicin (GM)	0/24	10/35 (28.6 %)	2/61 (3.3 %)	49 (80.3 %)	61
Levofloxacin (LEV)	0/34	0/26 (0 %)	0/60	60 (100 %)	60
Meropenem (MEM)	0/18	5/43 (11.6 %)	2/61 (3.3 %)	54 (88.5 %)	61
Total (%)	0/148	26/148 (17.6 %)	10/301 (3.3 %)	265 (88.1 %)	301
B					
Cefotaxime (CTX)	6/39 (15.4 %)	0/19	0/58	52 (89.7 %)	58
Ceftazidime (CAZ)	0/31	1/24 (4.2 %)	3/58 (5.2 %)	54 (93.1 %)	58
Gentamicin (GM)	0/25	8/33 (24.2 %)	1/60 (1.7 %)	51 (85 %)	60
Levofloxacin (LEV)	0/34	0/24	0/58	58 (100 %)	58
Meropenem (MEM)	0/16	6/42 (14.3 %)	2/58 (3.5 %)	50 (86.2 %)	58
Total (%)	6/145 (4.1 %)	15/142 (10.6 %)	6/292 (2.1 %)	265 (90.7 %)	292

^a^Using the results from the susceptibility testing of the bacteria by Vitek 2 as reference. In case of discrepancies between the two methods, the results for the susceptibility testing were confirmed by E-test. Data are numbers (with percentages) of bacterial isolates for which the antimicrobial susceptibility testing was concordant/correct or erroneous by PR1 (A) or PR2 (B)

**Table 4:** Antimicrobial susceptibility testing of Gram-negative nonfermenters from positive blood cultures by PR1 (A)^a^ or PR2 (B)^a^

Antimicrobial agent	No. of very major errors	No. of major errors	No. of minor errors	AST agreement	Total
A					
Amikacin (AMK)	0/3	0/10	0/13	13 (100 %)	13
Colistin (COL)	1/1 (100 %)	1/12 (8.3 %)	0/13	11 (84.6 %)	13
Gentamicin (GM)	1/2 (50 %)	1/11 (9.1 %)	0/13	11 (84.6 %)	13
Levofloxacin (LEV)	1/9 (11.1 %)	0/4	2/13 (15.4 %)	10 (76.9 %)	13
Total (%)	3/15 (20 %)	2/37 (5.4 %)	2/52 (3.8 %)	45 (86.5 %)	52
B					
Amikacin (AMK)	0/4	0/10	0	14 (100 %)	14
Colistin (COL)	0/0	0/14	0	14 (100 %)	14
Gentamicin (GM)	1/3 (33.3 %)	2/11 (18.2 %)	1/14 (7.1 %)	10 (71.5 %)	14
Levofloxacin (LEV)	0/9	0/5	2/14 (14.3 %)	12 (85.7 %)	14
Total (%)	1/16 (6.2 %)	2/40 (5 %)	3/56 (5.3 %)	50 (89.3)	56

^a^Using the results from the susceptibility testing of the bacteria by Vitek 2 as reference. In case of discrepancies, the results for the susceptibility testing were confirmed by E-test. Data are numbers (with percentages) of bacterial isolates for which the antimicrobial susceptibility testing was concordant/correct or erroneous by PR1 (A) or PR2 (B)

For Enterobacteriaceae (Table 3), categorical AST agreement was 88.1 by PR1, and 90.7 by PR2. By PR1, there were 26/148 (17.6 %) major errors, and 10/301 (3.3 %) minor errors; by PR2, there were 6/145 (4.1 %) very major errors, 15/142 (10.6 %) major errors, and 6/292 (2.1 %) minor errors. All the very major errors were observed for cefotaxime by PR2, whereas an AST agreement of 94.9 % was observed by PR1 for this antibiotic. On the contrary, ceftazidime reached significantly (*p* < 0.05) higher AST agreement by PR2 (93.1 %) than by PR1 (76.7 %). Of interest, AST agreement for levofloxacin was 100 % by both PR1 and PR2. Comparable AST agreement, below 90 %, was observed by PR1 and PR2 with gentamicin and meropenem.

For nonfermenters (Table 4), categorical AST agreement was 86.5 by PR1 and 89.3 by PR2. By PR1 there were 3/15 (20 %) very major errors, 2/37 (5.4 %) major errors, and 2/52 (3.8 %) minor errors; by PR2 there were 1/16 (6.2 %) very major errors, 2/40 (5 %) major errors, and 3/56 (5.3 %) minor errors. Of interest, AST agreement for amikacin was 100 % by both PR1 and PR2. AST agreement was 100 % also for colistin by PR2. AST agreement below 90 % was observed by PR1 and PR2 with gentamicin and levofloxacin, and with colistin by PR1.

### Direct AST of Gram-positive cocci

The AST results of 103 Gram-positive cocci were analysed, 76 (73.8 %) of which were coagulase-negative staphylococci, 17 (16.5 %) *S. aureus*, 8 (7.8 %) *Enterococcus* spp., and 2 (1.9 %) streptococci; no profiles were available for 1 *S. epidermidis* by PR1, and for 6 isolates (3 *S. hominis*, 1 *S. aureus*, 1 *Enterococcus faecium*, 1 *S. pneumoniae*) by PR2.

The antimicrobial susceptibility testing of Gram-positive cocci (Tables 5 and 6) was performed for a total of 305 and 290 isolate/antimicrobial agent combinations by PR1 and PR2, respectively. Discrepancies in the AST results between the direct and current methods were resolved by Etest, which revealed that the AST was correct by PR1 or PR2, but not by the current method, for 13 (4.3) and 14 (4.8 %) isolate/antimicrobial agent combinations, respectively, all of which regarding coagulase-negative staphylococci. The AST results by PR1 and PR2 and the percent errors are shown in Tables 5 and 6. Complete AST agreement was found for 81 (78.6 %) isolates by PR1 and 79 (76.7 %) by PR2, and for all *S. aureus* strains by PR1. Together, 93.1 and 93.8 % of the isolate/antimicrobial agent combinations showed concordant/correct AST results for Gram-positive cocci by PR1 and PR2, respectively. Of note, AST agreement with linezolid was 99 and 100 %, respectively, by PR1 and PR2. No significant difference in accuracy was found between PR1 and PR2 for Gram-positive cocci (0.5 < *P* < 0.9).

**Table 5:** Antimicrobial susceptibility testing of *Staphylococcus* species from positive blood cultures by PR1 (A)^a^ or PR2 (B)^a^

Antimicrobial agent	No. of very major errors	No. of major errors	No. of minor errors	AST Agreement	Total
A					
Cefoxitin (CFX)	1/63 (1.6 %)	5/29 (17.2 %)	2/92 (2.2 %)	84 (91.3 %)	92
Linezolid (LZ)	0/3	1/89 (1.1 %)	0/92	91 (98.9 %)	92
Teicoplanin (TEI)	0/0	6/92 (6.5 %)	3/92 (3.3 %)	83 (90.2 %)	92
Total (%)	1/66 (1.5 %)	12/210 (5.7 %)	5/276 (1.8 %)	258 (93.5 %)	276
B					
Cefoxitin (CFX)	3/61 (4.7 %)	4/28 (14.3 %)	3/89 (3.4 %)	79 (88.7 %)	89
Linezolid (LZ)	0/3	0/86	0/89	89 (100 %)	89
Teicoplanin (TEI)	0/0	7/89 (7.9 %)	0/89	82 (92.1 %)	89
Total (%)	3/64 (4.7 %)	11/203 (5.4 %)	3/267 (1.1 %)	250 (93.7 %)	267

^a^Using the results from the susceptibility testing of the bacteria by Vitek 2 as reference. In case of discrepancies, the results for the susceptibility testing were confirmed by E-test. Data are numbers (with percentages) of bacterial isolates for which the antimicrobial susceptibility testing was concordant/correct or erroneous by PR1 (A) or PR2 (B)

**Table 6:** Antimicrobial susceptibility testing of streptococci/enterococci from positive blood cultures by PR1 (A)^a^ or PR2 (B)^a^

Antimicrobial agent	No. of very major errors	No. of major errors	No. of minor errors	AST Agreement	Total
A					
Ampicillin (AMP)	1/5 (20 %)	0/4	0/9	8 (88.9 %)	9
Linezolid (LZ)	0/0	0/10	0/10	10 (100 %)	10
Teicoplanin (TEI)	0/1	2/9 (22.2 %)	0/10	8 (80 %)	10
Total (%)	1/6 (16.7 %)	2/23 (8.7 %)	0/29	26 (89.7 %)	29
B					
Ampicillin (AMP)	0/3	0/4	0/7	7 (100 %)	7
Linezolid (LZ)	0/0	0/8	0/8	8 (100 %)	8
Teicoplanin (TEI)	0/0	1/8 (12.5 %)	0/8	7 (87.5 %)	8
Total (%)	0/3	1/20 (5 %)	0/23	22 (95.7 %)	23

^a^Using the results from the susceptibility testing of the bacteria by Vitek 2 as reference. In case of discrepancies, the results for the susceptibility testing were confirmed by E-test. Data are numbers (with percentages) of bacterial isolates for which the antimicrobial susceptibility testing was concordant/correct or erroneous by PR1 (A) or PR2 (B)

For staphylococci (Table 5), by PR1 there were 1/66 (1.5 %) very major errors, 12/210 (5.7 %) major errors, and 5/276 (1.8 %) minor errors, and by PR2 there were 3/64 (4.7 %) very major errors, 11/203 (5.4 %) major errors, and 3/267 (1.1 %) minor errors. All the very major errors were observed with cefoxitin by both PR1 (one case) and PR2 (three cases). AST agreement was higher than 90 % for all the antibiotics tested by PR1, and for all but cefoxitin by PR2. Complete AST agreement was observed with linezolid by PR2.

For streptococci/enterococci (Table 6), representing only 9.7 % of all the Gram-positive cocci tested, by PR1 there were 1/6 (16.7 %) very major errors, and 2/23 (8.7 %) major errors, and by PR2 there was 1/20 (5 %) major error. Complete agreement for all isolates was observed with linezolid by both PR1 and PR2, and with ampicillin by PR2. AST agreement below 90 % was obtained with teicoplanin by both PR1 and PR2, and with ampicillin by PR1.

## Discussion

In the last few years, the rapid development of MALDI-TOF MS technology has allowed rapid bacterial species identification directly from positive BC [10, 11, 24–30].

In the present study, direct MALDI-TOF analysis was used for rapid identification of bacteria from monomicrobial BC, as previously described [11]. Concordant identification of Gram-negative bacteria and Gram-positive cocci by the direct MALDI-TOF method compared to the current method was 97.5 % and 96.1 %, respectively, supporting that direct MALDI-TOF can be used efficiently for ID of bacteria in positive BC [11, 27, 29, 30]. However, it should be noted that only score values ≥ 2.000 are considered as probable species identification. As we observed in a previous study, our data strongly suggest that cut-off values could be lowered down to 1.4 without compromising accuracy [11]. Similar observations have been reported also by other authors [28, 30, 31]. Therefore, the availability of rapid ID results by this method allowed proceeding with the selection of the proper antibiotic panel for direct AST on the basis of those available for this new system. The main finding from the present study is that the proposed method for direct AST might be used to provide clinicians with reliable AST results in a few hours. The AST results of Gram-negative bacteria obtained by the two different protocols, PR1 and PR2, were concordant/correct with the results obtained by the current method for 87.8 and 90.5 % of the isolate/antimicrobial agent combinations, respectively. For Gram-negative bacteria, levofloxacin showed an agreement in clinical categories >90 % by both PR1 and PR2, with <3 % very major errors and <7 % major and minor errors in combination, thus meeting the selection criteria for an antimicrobial susceptibility testing proposed by Jorgensen [32].

For Enterobacteriaceae, there was 100 % categorical agreement for levofloxacin by both PR1 and PR2, 94.9 % for cefotaxime by PR1 and 93.1 % for ceftazidime by PR2. The lowest categorical agreement was found for ceftazidime by PR1 (significantly lower than by PR2). Unfortunately, meropenem and ceftazidime tests were not available for nonfermenters. Although the number of nonfermenters tested was low, 100 % categorical agreement was observed with amikacin by PR1 and PR2, and with colistin by PR2. Altogether, no significant difference in accuracy was found for Gram-negative bacteria between PR1 and PR2.

The AST results of Gram-positive cocci by PR1 and PR2 were concordant/correct with the results obtained by the current method for 93.1 and 93.8 % of the isolate/antibiotic combinations, respectively. Cefoxitin (91.3 %) and linezolid (99.0 %) by PR1, and linezolid (100 %) and teicoplanin (91.8 %) by PR2 showed an agreement in clinical categories >90 %, with linezolid meeting the selection criteria proposed by Jorgensen, both by PR1 and PR2. With the only exception of cefoxitin (88.7 %) by PR2, there was a categorical agreement >90 % for all the antimicrobial agents tested on staphylococci by both PR1 and PR2. Optimal results were obtained for *S. aureus* isolates by PR1, with 17/17 isolates showing complete AST agreement with the current method. Although the number of streptococci/enterococci tested was low, 100 % categorical agreement was observed with linezolid, by PR1 and PR2, and ampicillin by PR2. In the overall, no significant difference in accuracy was found between PR1 and PR2 for Gram-positive cocci. The time required by PR1 from blood culture positivity to AST results is about 4 h, including about 30 min to prepare the bacterial suspension and obtaining ID results, and a three-hour incubation for most of the tested antibiotics. For antibiotics requiring a five-hour incubation, 6 h are needed to provide AST results by PR1. The time required by PR2 from blood culture positivity to AST results is more variable, depending on the time required to reach the 0.5 McF suspension (1–4 h) and the whole procedure may take up to 9 h. On the bases of these observations, PR1 is to be preferred to PR2.

## Conclusions

The most relevant conclusion from the present study is that the proposed method allows to yield rapid and reliable AST results, which often is of vital importance to either confirm or streamline antibiotic therapy for patients suffering from BSI. In addition, the earlier administration of appropriate antimicrobial therapy will also reduce the emergence of antimicrobial-resistant strains. The results were particularly favorable for Gram-positive cocci, which often yield less accurate results than Gram-negative bacteria when tested directly from positive BC. In particular, 100 % categorical agreement was found with levofloxacin for Enterobacteriaceae by both PR1 and PR2, and 99.0 and 100 % categorical agreement was observed with linezolid for Gram-positive cocci by PR1 and PR2, respectively. A limitation of this study is that times to result were not accurately measured. Further studies involving a higher number of blood samples and antibiotics are needed to fully evaluate the potential of this new method. Important advancement in patient care in terms of clinical and financial benefits is to be expected by its implementation in clinical microbiological laboratories.

## Abbreviations

AST, antimicrobial susceptibility testing; BC, blood cultures; BSI, bloodstream infections; ID, identification; MALDI-TOF, matrix-assisted laser desorption/ionization time-of-flight; PR1, protocol 1; PR2, protocol 2; MIC, minimum inhibitory concentration

## Additional file

Additional file 1: Table S1. Number of susceptible, intermediate and resistant isolates for each species/antimicrobial agent combination according to the current method: Enterobacteriaceae (A), Gram-negative nonfermenters (B), staphylococci (C), and streptococci-enterococci (D). (DOCX 17 kb)
