# Supplementary material for: A new rapid method for direct antimicrobial susceptibility testing of bacteria from positive blood cultures
Source: BMC Microbiol. 2016 Aug 12;16:185. doi: 10.1186/s12866-016-0805-5 (PMC4982226; doi:10.1186/s12866-016-0805-5)
Supplement: Additional file 1: Table S1. — Number of susceptible, intermediate and resistant isolates for each species/antimicrobial agent combination according to the current method: Enterobacteriaceae (A), Gram-negative nonfermenters (B), staphylococci (C), and streptococci-enterococci (D). (DOCX 17 kb) [file 12866_2016_805_MOESM1_ESM.docx]

**Table AM-1**. Number of susceptible, intermediate and resistant strains for each isolate/antimicrobial agent combination by the current method. Enterobacteriaceae (A), Gram-negative nonfermenters (B), staphylococci (C), and streptococci/enterococci (D).

R: resistant; S: sensitive; I: intermediate

A

| **Species** | **CTX** | | | **CAZ** | | | **GM** | | | **LEV** | | | **MEM** | | |
| --- | --- | --- | --- | --- | --- | --- | --- | --- | --- | --- | --- | --- | --- | --- | --- |
|  | **R** | **S** | **I** | **R** | **S** | **I** | **R** | **S** | **I** | **R** | **S** | **I** | **R** | **S** | **I** |
| *E. coli* | 12 | 11 | 0 | 9 | 13 | 1 | 19 | 5 | 0 | 12 | 11 | 0 | 1 | 23 | 0 |
| *K. pneumoniae* | 20 | 5 | 0 | 19 | 4 | 2 | 3 | 20 | 2 | 20 | 5 | 0 | 16 | 9 | 0 |
| *Enterobacter* spp. | 5 | 1 | 0 | 4 | 2 | 0 | 1 | 5 | 0 | 2 | 4 | 0 | 1 | 5 | 0 |
| *M. morganii* | 2 | 0 | 0 | 0 | 2 | 0 | 0 | 2 | 0 | 0 | 2 | 0 | 0 | 2 | 0 |
| *S. marcescens* | 2 | 0 | 0 | 0 | 2 | 0 | 0 | 2 | 0 | 0 | 2 | 0 | 0 | 2 | 0 |
| *Salmonella sp* | 0 | 1 | 0 | 0 | 1 | 0 | 1 | 0 | 0 | 0 | 1 | 0 | 0 | 1 | 0 |
| *P. mirabilis* | 1 | 0 | 0 | 1 | 0 | 0 | 1 | 0 | 0 | 1 | 0 | 0 | 0 | 1 | 0 |
| *R. ornithinolytica* | 0 | 1 | 0 | 0 | 1 | 0 | 0 | 1 | 0 | 0 | 1 | 0 | 0 | 1 | 0 |
| sub-total | **42** | **19** | **0** | **33** | **25** | **3** | **25** | **35** | **2** | **35** | **26** | **0** | **18** | **44** | **0** |
| total | 61 | | | 61 | | | 62 | | | 61 | | | 62 | | |

B

| **Species** | **AMK** | | | **COL** | | | **GM** | | | **LEV** | | |
| --- | --- | --- | --- | --- | --- | --- | --- | --- | --- | --- | --- | --- |
|  | **R** | **S** | **I** | **R** | **S** | **I** | **R** | **S** | **I** | **R** | **S** | **I** |
| *P. aeruginosa* | 2 | 10 | 0 | 0 | 12 | 0 | 1 | 11 | 0 | 8 | 4 | 0 |
| *A. baumannii* | 2 | 1 | 0 | 0 | 3 | 0 | 2 | 1 | 0 | 2 | 1 | 0 |
| *S. maltophilia* | 2 | 0 | 0 | 1 | 1 | 0 | 2 | 0 | 0 | 2 | 0 | 0 |
| *A. ursingii* | 0 | 1 | 0 | 0 | 1 | 0 | 0 | 1 | 0 | 0 | 1 | 0 |
| sub-total | **6** | **12** | **0** | **1** | **17** | **0** | **5** | **13** | **0** | **12** | **6** | **0** |
| total | 18 | | | 18 | | | 18 | | | 18 | | |

C

| **Species** | **CFX** | | | **LZ** | | | **TEI** | | |
| --- | --- | --- | --- | --- | --- | --- | --- | --- | --- |
|  | **R** | **S** | **I** | **R** | **S** | **I** | **R** | **S** | **I** |
| *S. epidermidis* | 35 | 7 | 0 | 2 | 40 | 0 | 0 | 42 | 0 |
| *S. aureus* | 6 | 11 | 0 | 0 | 17 | 0 | 0 | 17 | 0 |
| *S. hominis* | 7 | 5 | 0 | 0 | 12 | 0 | 0 | 12 | 0 |
| *S. capitis* | 8 | 3 | 0 | 1 | 10 | 0 | 0 | 11 | 0 |
| *S. haemolyticus* | 6 | 0 | 0 | 0 | 6 | 0 | 0 | 6 | 0 |
| *S. warneri* | 0 | 2 | 0 | 0 | 2 | 0 | 0 | 2 | 0 |
| *S. pettenkoferi* | 1 | 0 | 0 | 0 | 1 | 0 | 0 | 1 | 0 |
| *S. sciuri* | 1 | 0 | 0 | 0 | 1 | 0 | 0 | 1 | 0 |
| *S. lugdunensis* | 0 | 1 | 0 | 0 | 1 | 0 | 0 | 1 | 0 |
| sub-total | **64** | **29** | **0** | **3** | **90** | **0** | **0** | **93** | **0** |
| total | **93** | | | **93** | | | **93** | | |

D

| **Species/genus** | **AMP** | | | **LZ** | | | **TEI** | | |
| --- | --- | --- | --- | --- | --- | --- | --- | --- | --- |
|  | **R** | **S** | **I** | **R** | **S** | **I** | **R** | **S** | **I** |
| *E. faecium* | 4 | 0 | 0 | 0 | 4 | 0 | 1 | 3 | 0 |
| *E. faecalis* | 0 | 3 | 0 | 0 | 3 | 0 | 0 | 3 | 0 |
| *E. casseliflavus* | 0 | 1 | 0 | 0 | 1 | 0 | 0 | 1 | 0 |
| *Streptococcus spp* | 1 | 0 | 0 | 0 | 2 | 0 | 0 | 2 | 0 |
| sub-total | **5** | **4** | **0** | **0** | **10** | **0** | **1** | **9** | **0** |
| total | 9 | | | 10 | | | 10 | | |

CTX: cefotaxime; CAZ: ceftazidime; GM: gentamicin; LEV: levofloxacin; MEM: meropenem;

AMK: amikacin; COL: colistin; CFX: cefoxitin; LZ: linezolid; TEI: teicoplanin; AMP: ampicillin.
